# Application of transfer learning for cancer drug sensitivity prediction

**DOI:** 10.1186/s12859-018-2465-y

**Published:** 2018-12-28

**Authors:** Saugato Rahman Dhruba, Raziur Rahman, Kevin Matlock, Souparno Ghosh, Ranadip Pal

**Affiliations:** 1 Department of Electrical and Computer Engineering, Texas Tech University, 1012 Boston Ave, Lubbock, 79409 TX USA; 2 Department of Mathematics and Statistics, Texas Tech University, 1108 Memorial Circle, Lubbock, 79409 TX USA

**Keywords:** Drug sensitivity prediction, Pharmacogenomic studies, CCLE, GDSC, Transfer learning, Nonlinear mapping, Latent variable, Cost optimization

## Abstract

**Background:**

In precision medicine, scarcity of suitable biological data often hinders the design of an appropriate predictive model. In this regard, large scale pharmacogenomics studies, like CCLE and GDSC hold the promise to mitigate the issue. However, one cannot directly employ data from multiple sources together due to the existing distribution shift in data. One way to solve this problem is to utilize the transfer learning methodologies tailored to fit in this specific context.

**Results:**

In this paper, we present two novel approaches for incorporating information from a secondary database for improving the prediction in a target database. The first approach is based on latent variable cost optimization and the second approach considers polynomial mapping between the two databases. Utilizing CCLE and GDSC databases, we illustrate that the proposed approaches accomplish a better prediction of drug sensitivities for different scenarios as compared to the existing approaches.

**Conclusion:**

We have compared the performance of the proposed predictive models with database-specific individual models as well as existing transfer learning approaches. We note that our proposed approaches exhibit superior performance compared to the abovementioned alternative techniques for predicting sensitivity for different anti-cancer compounds, particularly the nonlinear mapping model shows the best overall performance.

**Electronic supplementary material:**

The online version of this article (10.1186/s12859-018-2465-y) contains supplementary material, which is available to authorized users.

## Background

A consistent challenge in precision medicine is to design appropriate models for predicting the sensitivity of a tumor to an anti-cancer compound with high accuracy. In this aspect, large-scale pharmacogenomic studies of cancer genomes have provided unprecedented insights for studying anti-cancer therapeutics to determine putative prediction of drug sensitivity. The Genomics of Drug Sensitivity in Cancer (GDSC) [1] of the Cancer Genome Project and the Cancer Cell Line Encyclopedia (CCLE) [2] from the Broad Institute are two such studies where drug sensitivity profiles and genomic information across hundreds of compounds and cancer cell lines have been systematically gathered. There exists significant overlaps between the two databases which can further be utilized in designing more accurate sensitivity predictive models. Biological data for designing suitable predictive models are frequently scarce and therefore the availability of a secondary dataset often holds the promise for a better model development. However, majority of the machine learning approaches used in drug sensitivity prediction follow the inherent assumption that both training data and test data are in the same feature space with the same distribution. But, when training and test data, despite being in the same feature space, exhibit different distributions, one need to take the distribution shift into account. This is where transfer learning (TL) methodologies come into play [3].

Often in TL environment, the source and target domains can be considered as linked subspaces as part of a high-level common domain space [4]. We, therefore need to assume that there exists some consistency between the different datasets to be utilized in TL. Haibe-Kains et al. [5] at first pointed out that, although the gene expression from CCLE and GDSC databases are well correlated between themselves, unexpectedly the measured pharmacological drug responses using common estimators such as IC_50_ and the area under the curve (AUC) measures are highly discordant. In response, the CCLE and GDSC investigators performed their own analysis [6] and presented results opposing the conclusions in [5]. They pointed out that in majority of the drugs, the exhibited AUC and IC_50_ distributions are dominated by drug insensitive lines with a much smaller number of outliers, and postulated that the differences in cell line biology between studies have resulted in the poor correlation. Considering these facts, they have demonstrated significant improvement in correlation between most of the drugs. In any event, the fact that both the databases are providing information about the same biological process, make them suitable candidates for applying transfer learning methodologies.

In case of inconsistent data with different distributions for training and test sets, various TL approaches [3] have been attempted for dataset shift. Unsupervised methods such as INSPIRE (INferring Shared modules from multiPle gene expREssion datasets) [7] is primarily focused on the expression datasets to extract a low-dimensional representation and predicts tumor phenotypes using regularized regression approaches. Inductive transfer learning (ITL) approaches, as in [8], tackle the issue of prediction for scarce primary data using a secondary dataset through importance sampling *i.e.*, reweighting the secondary distribution to the primary. While the primary data size is assumed to be significantly smaller than secondary data, for large number of unlabeled data, one has to adapt to covariate shift along with ITL. Boosting based approaches such as Dynamic-TrAdaBoost [9] applies ensemble methods to both source and target instances and then employs an update mechanism incorporating only the source instances useful for target task, with an additional dynamic correction factor. Kernel based ITL methods [10, 11] focus on finding an appropriate kernel for the newly available data, modeling the difference with existing data as a problem of finding the suitable bias.

**Fig. 1:**
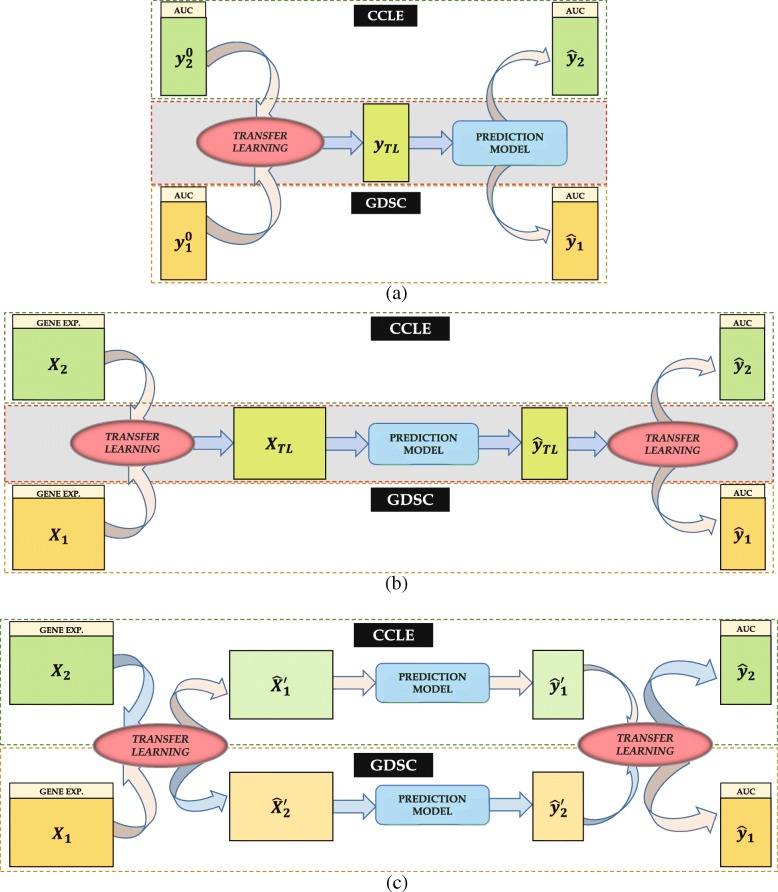
Illustration of the proposed transfer learning approaches: (**a**) Optimization based TL, applied on output only, (**b**) Optimization based TL, applied on both input and output, and (**c**) Domain transfer TL, applied on both input and output

The previous approaches for transfer learning work well under the assumption that the datasets are closely related (such as 9 ovarian cancer datasets in INSPIRE) and the number of samples are significantly larger than the number of features (*n*>*p*). However, the scenario is frequently reversed in the case of genomic (or proteomic) data *i.e.*, we usually have tens of thousands of genes and a small number of cell lines. Additionally, the previous methods for TL often involve removing the distribution shift via weighting without any explicit domain transfer. In our work, we have proposed two different TL approaches that consider mapping the data from two different databases to either a common space or to each other’s domain, inherently taking care of the *n*<<*p* problem. The inherent assumptions here for each pair of similar datasets from CCLE and GDSC are – (i) The datasets are monotonically changing in the same direction, and (ii) There exists a functional relationship between them. To build an appropriate prediction model, we utilize the gene expression as the predictors and the drug sensitivity (specifically AUC) as the output. Considering the application of TL on these datasets, the proposed approaches in this paper can be classified into two categories, as illustrated in Fig. 1.

- Cost optimization based approach where we employ latent variable models to extract the underlying variables between different datasets. In this case, TL can be applied to only the output (Fig. 1(a)), as in parameter transfer approach [12, 13] or to both model input and output (Fig. 1(b)), as in [14, 15].
- Domain transfer approach where we design maps between databases to transfer data from primary domain to secondary and utilize the secondary data to improve the prediction model. Here, TL is applied to both input and output (Fig. 1(c)), as in instance transfer approach [14, 15].

To summarize, the key contributions of the paper is – we have implemented two TL based approach, where the target (primary) data is either transferred to a common latent variable space along with the source (secondary) data, or to the source domain through nonlinear mapping to improve the prediction of limited primary data employing the available secondary data.

## Results

To evaluate the performance of our transfer learning algorithms, we have initially retrieved the data common to both CCLE and GDSC. From GDSC (*v*6.0) and CCLE, there are 15,664 common genes available in 623 common cell lines along with 15 common drugs. We have performed a drug-wise analysis and found that the number of cell lines decreases from 623 after incorporating the available drug sensitivity values, resulting in datasets with cell lines between 91−310 along with 15,664 genes and corresponding sensitivity measures. For analysis involving gene expression, we have used ReliefF [16] to select the top 200 genes from each dataset and taken the intersection as the final feature set. For drug sensitivity measure, we have used the AUC values as they have more concordance between databases (median *ρ*_*s*_=0.34) than IC_50_ (median *ρ*_*s*_=0.28) [5]. Note that in spite of our discussion on inconsistencies between databases, the main goal here is to consider the scenario where a small portion of database 1 (*i.e.*, GDSC) is available while data for the entire database 2 (*i.e.*, CCLE) is available and we would like to use database 2 to improve the prediction performance for the rest of database 1. Thus, for evaluation, we will use the GDSC experimental AUCs as the *gold standard* and compare with the predicted AUCs.

### Latent variable cost optimization approach

**Fig. 2:**
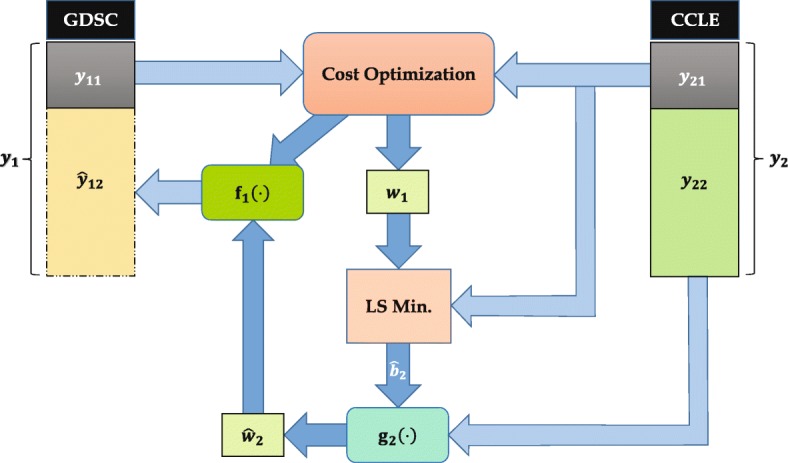
Illustration of Latent Regression Prediction. Here, unknown set of GDSC AUC values, *y*_12_, is predicted using the underlying latent vector, *w*_2_, calculated from corresponding CCLE AUC set, *y*_22_

**Fig. 3:**
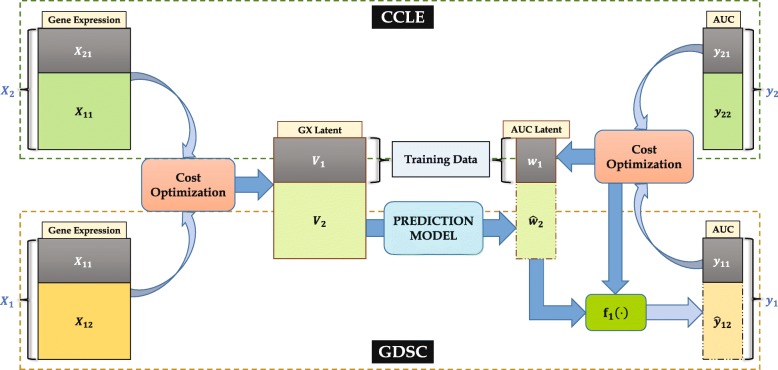
Illustration of Latent-Latent Prediction. Here, unknown set of GDSC AUC values *y*_12_ is predicted using the underlying latent variables *V* and *w*_1_, calculated from *X*_1_,*X*_2_ and *y*_11_,*y*_21_. *V*_1_ & *w*_1_ are used for training while *V*_2_ is used to predict *w*_2_ and then *y*_12_

We have performed drug sensitivity prediction using the three latent variable cost optimization based approaches – Latent Regression Prediction (LRP), Latent-Latent Prediction (LLP), Combined Latent Prediction (CLP) (described in the “Methods” section) for 7 common drugs with sufficient cell lines (*n*>200). For each method, subsets of 50 randomly chosen GDSC cell lines (*X*_11_ & *y*_11_ in Figs. 2 & 3) are used for the cost optimization in training and the rest (*y*_12_) are predicted along with the known CCLE data (*X*_2_ & *y*_2_ in Figs. 2 & 3). Table 1 illustrates the comparison of prediction performance for all three methods with *Direct prediction (DP)* for *K*-fold cross-validation, where DP is defined as training on the 50 available cell lines and predicting for the rest. Here, the number of folds is found as $K = \frac {n}{50}$, where 1 fold (containing ∼ 50 samples) is used for training and the remaining (*K*−1) folds are used for testing.

**Table 1:** Comparison of *K*-fold cross-validation performance for 4 GDSC drug sensitivity prediction approaches – Latent Regression Prediction (LRP), Latent-Latent Prediction (LLP), Combined Latent Prediction (CLP) and Direct Prediction (DP), using data from CCLE

Drug	Pearson Correlation				NRMSE			
	LRP	LLP	CLP	DP	LRP	LLP	CLP	DP
17-AAG	0.5441	0.4691	**0.6382**	0.4591	0.2117	0.2147	**0.1930**	0.2164
AZD6244	0.3988	0.4155	**0.4524**	0.4008	0.1833	0.1718	**0.1684**	0.1703
Nilotinib	**0.9053**	0.3886	0.8768	0.4524	**0.0728**	0.1295	0.0888	0.1242
Nutlin-3	0.4093	0.5473	**0.5646**	0.5108	0.1965	0.1756	**0.1745**	0.1799
PD-0325901	0.6448	0.4502	**0.6606**	0.4465	0.1614	0.1870	**0.1585**	0.1878
PD-0332991	0.2497	0.0912	**0.2540**	0.0884	0.1695	0.1729	**0.1672**	0.1733
PLX4720	0.5682	0.5040	**0.6384**	0.5001	0.1237	0.1290	**0.1173**	0.1291

Bold values indicate the best performance

### Domain transfer approach

**Fig. 4:**
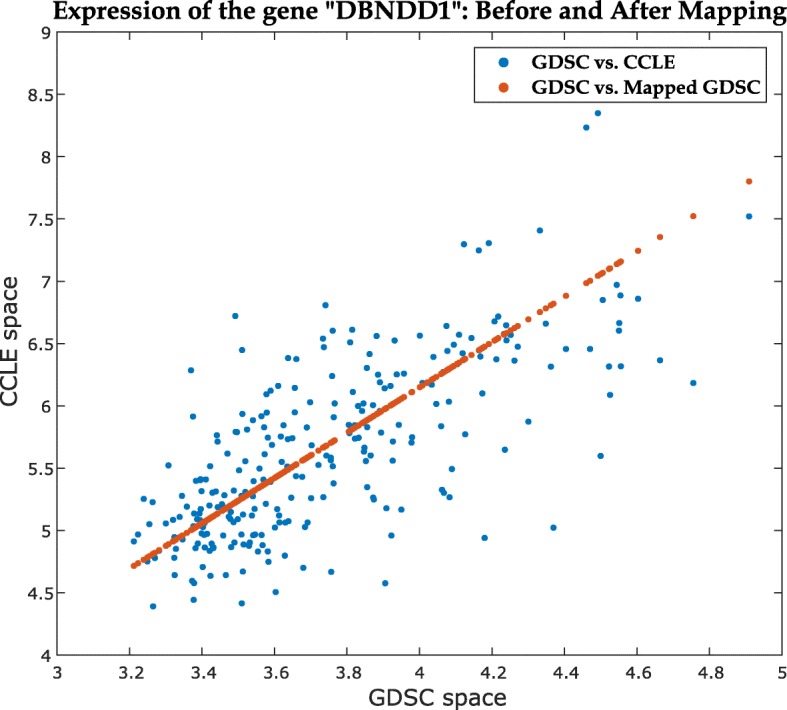
Scatter plot of gene expression association between GDSC and CCLE spaces before and after applying the polynomial mapping for the gene “DBNDD1”

**Fig. 5:**
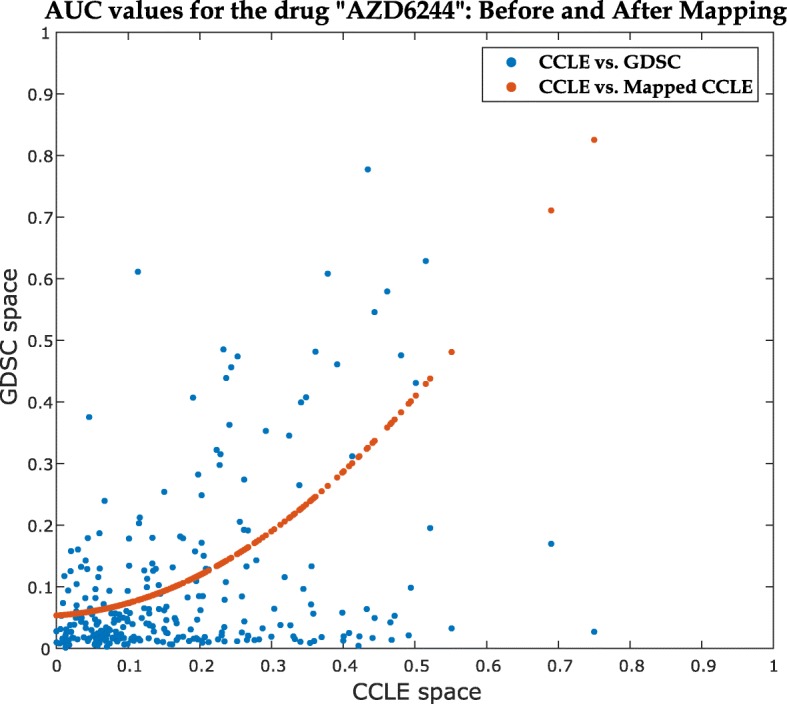
Scatter plot of AUC association between GDSC and CCLE spaces before and after applying polynomial mapping for the drug “AZD6244” (*ρ*_*s*_=0.26)

**Fig. 6:**
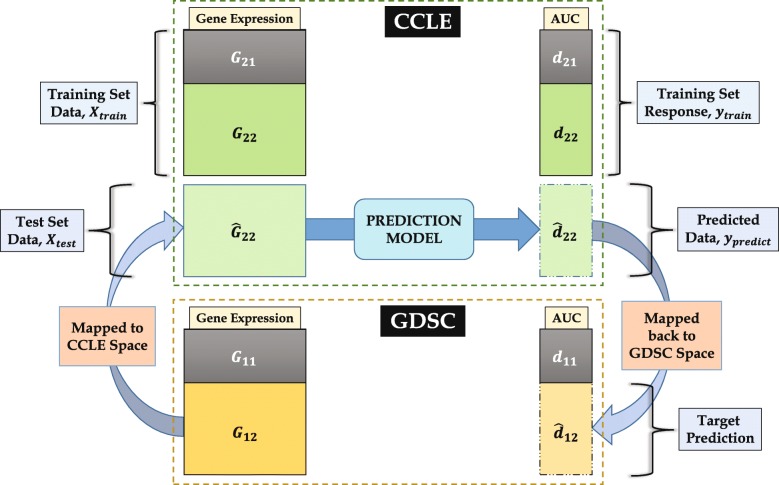
Illustration of drug sensitivity prediction for GDSC using the nonlinear mapping between CCLE and GDSC

We have performed the Mapped Prediction (MP) approach (described in the “Methods” section) for predicting GDSC sensitivities for 7 common drugs with sufficient cell lines (*n*>200) and different levels of database consistency. Figure 4 demonstrates the effect of first-order polynomial mapping for a representative gene expression set, while Fig. 5 illustrates the effect of second-order polynomial mapping for a representative drug sensitivity vector. Again, we used random subsets of 50 cell lines (*G*_11_,*d*_11_ & *G*_21_,*d*_21_ in Fig. 6) to retrieve the mapping functions and sensitivities for the rest (*d*_12_) are predicted using the known CCLE data (*G*_22_,*d*_22_). Table 2 shows the comparison of prediction performance for MP approach for all 7 drugs with two other methods – Direct Prediction (DP) and *CCLE model prediction (CP)* for *K*-fold cross-validation, as defined above (*i.e.*, $K = \frac {n}{50}$ and 1 fold is used for training and (*K*−1) folds for testing). For CP approach, the model is built using the available CCLE data directly and prediction is performed using the GDSC expression data. For prediction of AUC values using gene expression data, we have used a Bias-corrected Random Forest (BC-RF) [17–19] model.

**Table 2:** Comparison of *K*-fold cross-validation performance for three GDSC drug sensitivity prediction approaches – Mapped Prediction (MP), CCLE model Prediction (CP) and Direct Prediction (DP) using data from CCLE

Drug	Pearson Correlation			NRMSE		
	MP	CP	DP	MP	CP	DP
17-AAG	**0.6062**	0.4354	0.4591	**0.2112**	0.3073	0.2164
AZD6244	**0.4692**	0.3580	0.3579	**0.1683**	0.2173	0.1743
Nilotinib	**0.8698**	0.7957	0.4524	**0.1093**	0.1323	0.1242
Nutlin-3	**0.5606**	0.3102	0.5114	0.1852	0.2180	**0.1808**
PD-0325901	**0.6132**	0.5731	0.4224	**0.1689**	0.1875	0.1865
PD-0332991	**0.0923**	0.0305	0.0802	**0.1748**	0.1764	0.1755
PLX4720	**0.6335**	0.6135	0.5001	**0.1242**	0.159	0.1291

Bold values indicate the best performance

## Discussion

From Table 1, it is evident that the CLP method yields the best performance. Additionally, even though the LLP method often yield better results than DP, it frequently underperforms than LRP. Overall, 6 drugs out of 7 yield the best performance for CLP method while only Nilotinib performs the best with LRP. The prediction performance is similar in the reverse direction (*i.e.*, CCLE as the primary set and GDSC as secondary) where 5 out of 7 drugs show best performance for CLP.

For the Domain Transfer approach, it is evident from Table 2 that the MP approach performs significantly better than the both CP and DP. Furthermore, the performance of the CP approach is much worse compared to either MP or DP, which can be attributed to the existing distribution shift between CCLE and GDSC data in general. Note that among the 7 drugs, 17-AAG and PD-0325901 has moderate concordance (0.5≤*ρ*_*s*_<0.6) while AZD6244, Nutlin-3 and PD-0332991 have poor concordance (*ρ*_*s*_<0.4) between databases. For PLX4720 and Nilotinib, there exist moderate to high consistency in terms of Pearson correlation (*ρ*=0.57 and *ρ*=0.88 respectively), although the rank correlation is low (*ρ*_*s*_=0.29 and *ρ*_*s*_≈0.1 respectively). We have also implemented a model that uses the ensemble of available CCLE and GDSC data directly for training and predicts for the unlabeled GDSC expression data, referred as the *Combined Model Prediction*. An additional section provides a detailed description and comparative analysis of this model with the MP approach [see Additional file 1].

***Comparison with inductive transfer learning***

We have compared the results from the Mapped Prediction approach with an existing transfer learning approach, namely the *Importance-weighted Direct Inductive Transfer Learning (DITL)* proposed by Garcke et al. [8]. In DITL, the primary and secondary datasets are assumed to be related in a way so that in some parts of the domain, the two distributions can be similar, and therefore, one can employ the secondary dataset with primary via importance sampling (*i.e.*, reweighting the secondary distribution to the primary so that the secondary data points with positive effect on primary data will have greater weights). For prediction, DITL uses weighted Kernel Ridge regression (KRR) with *Gaussian* kernels, dubbing the whole approach as DITL-KRR [8]. Table 3 shows the comparison of prediction performance for DITL-KRR approach with MP and DP approaches for 4 representative drugs. Unlike the MP approach, DITL follows the *n*>*p* assumption of machine learning and therefore, we used the intersection of top 50 genes from both datasets as the feature set while 50 cell lines were used for training. From Table 3, we can conclude that MP has a superior performance compared to the other approaches even when the number of features (therefore, information) is reduced to < 50.

**Table 3:** Comparison of prediction performance for DITL-KRR approach with Mapped Prediction (MP) and Direct Prediction (DP) approaches for 4 common drugs

Drug	Number of features	Pearson Correlation			NRMSE		
		MP	DP	DITL-KRR	MP	DP	DITL-KRR
17-AAG	47	**0.6319**	0.4749	-0.2885	**0.1942**	0.2167	0.4056
AZD6244	49	**0.4407**	0.4016	-0.1468	**0.1554**	0.1570	0.2042
Nilotinib	35	**0.9338**	0.4674	-0.1701	**0.1003**	0.1257	0.1410
Nutlin-3	48	**0.5921**	0.5207	-0.1500	**0.1881**	0.1903	0.2697

Here, intersection of top 50 genes is taken as the feature set. Bold values indicate the best performance

## Conclusions

In precision medicine, data from multiple large pharmacological studies can be utilized to design better predictive models. In this regard, transfer learning is employed to eliminate the distribution shift between the primary and secondary datasets. In this paper, we have proposed two different TL approaches to incorporate data from two large studies *i.e.*, CCLE and GDSC for designing a better predictive model. In the first approach, we have used a latent variable approach and then optimized the appropriate cost functions to get a pertinent prediction model. The second method uses a nonlinear mapping between both genomic and sensitivity data to transfer the primary data to secondary domain space and perform prediction utilizing the secondary datasets. Both methods show marked improvement in drug sensitivity prediction compared to direct prediction and existing TL approaches, while the mapping approach shows the best overall performance.

We have faced a couple of issues during implementation. The LRP approach utilizes the underlying latent variable between the sensitivity datasets and generate the latent variable corresponding to unknown primary sensitivity data. However, to do so, it uses the available secondary data inferring that the prediction can be only performed for matched pair of datasets. Although the LLP approach overcomes this limitation, it often underperforms than LRP. In Table 4, we have presented the applicability of the sensitivity prediction approaches discussed in this paper for matched vs. unmatched pairs of datasets.

**Table 4:** Applicability of Drug Sensitivity Prediction approaches for Matched and Unmatched Pairs of sets between Databases

Prediction Approach	Applicability	
	Matched	Unmatched
Direct Prediction	Yes	Yes
Latent Regression Prediction	Yes	No
Latent-Latent Prediction	Yes	Yes
Combined Latent Prediction	Yes	No
Mapped Prediction (Domain Transfer)	Yes	Yes
Direct Inductive Transfer Learning	Yes	Yes

Furthermore, in Mapped Prediction, drug sensitivity mapping between databases using polynomials is drug-dependent and thus vulnerable to a user-fault. One potential new step can be modeling the map to be robust against the outliers. Another development can be investigating the effect of model stacking using the proposed approaches.

## Methods

### Latent variable cost optimization approach

In this section, our goal is to analyze the transfer learning approach from the viewpoint of a cost function optimization. Here, the assumption is that– if there exists such a way to transfer data from both CCLE and GDSC to a common space, then the information available in both databases can be incorporated together to result in a better overall performance [3]. Therefore, it can be inferred that in a suitable common space, the individual concordance between the common set (*i.e.*, underlying latent variable) and each dataset will be maximized and the reconstruction errors from the common set will be minimized. This is the rationale behind the cost function optimization approach.

#### Drug sensitivity prediction via cost optimization of sensitivity data

In this section, we have deployed cost function optimization of CCLE and GDSC sensitivity data to utilize the underlying latent vector for improving the sensitivity prediction to an anti-cancer drug. The hypothesis is that if both CCLE and GDSC sensitivity vectors can be represented as functions of a common latent variable, then this variable can be utilized along with a known set of CCLE sensitivity values to predict the unknown GDSC sensitivity or vice versa. This approach is regarded as the *Latent Regression Prediction (LRP)*, as the final prediction is performed using a regression model on the latent vector. For this method, only the drug sensitivity values (namely AUC) from the two databases are employed without any use of genomic characteristics data. Figure 2 illustrates the use of LRP method for drug sensitivity prediction. Assume that only a small portion, $\phantom {\dot {i}\!}(y_{11})_{n_{1} \times 1}$ of GDSC AUC set, (*y*_1_)_*n*×1_, is known, where *n*_1_<*n*. Then, the corresponding AUC set, $\phantom {\dot {i}\!}(y_{21})_{n_{1} \times 1}$, in CCLE can be used with *y*_11_ to perform a cost optimization to retrieve the optimum weight vector *c* for the latent variable, $\phantom {\dot {i}\!}(w_{1})_{n_{1} \times 1}$, as follows (An additional section provides the detailed development of the cost function [see Additional file 1])

1 $$\begin{array}{*{20}l}  \min_{c} &{\frac{\left\| y_{11} - W_{1} a_{1} \right\|_{2}^{2} + \left\| y_{21} - W_{1} a_{2} \right\|_{2}^{2}} {\rho(y_{11}, w_{1}) + \rho(y_{21}, w_{1})}}\\& \quad\quad\text{subject to} \ \; \begin{array}{ll} -1 \leq c_{0} \leq 1, \\ 0 \leq c_{1}, c_{2} \leq 1, \\ c_{1} + c_{2} = 1 \end{array} \end{array} $$

where $ W_{1} = \left [\begin {array}{ll} \vec {1} & w_{1} \end {array}\right ]$, $c = \left [\begin {array}{lll} c_{0} & c_{1} & c_{2} \end {array}\right ]^{T}$and $\vec {1}$ denotes a vector-of-one. Here, *w*_1_ is the latent vector corresponding to *y*_11_ & *y*_21_ and assuming linear relationships, *c*_1_ & *c*_2_ are the weights of *y*_11_ & *y*_21_ in *w*_1_ (while *c*_0_ is the offset), defined as

2 $$\begin{array}{*{20}l} w_{1} = c_{0} + c_{1} y_{11} + c_{2} y_{21} + \varepsilon = \left[\begin{array}{lll} \vec{1} & y_{11} & y_{21} \end{array}\right] c + \varepsilon \end{array} $$

Now, *a*_1_ & *a*_2_ are the regression coefficients for reconstruction of *y*_11_ & *y*_21_ from *w*_1_ and can be obtained from the Least Squares (LS) minimizations of the reconstruction errors (*ε*).

3 $$ \begin{aligned} y_{11} = \mathrm{f}_{1} (w_{1}) = W_{1} a_{1} + \varepsilon_{1} \\ y_{21} = \mathrm{f}_{2} (w_{1}) = W_{1} a_{2} + \varepsilon_{2} \\ \qquad\text{where}\, a_{1} = \left[\begin{array}{cc} a_{10} \\ a_{11} \end{array}\right] \!, \: a_{2} = \left[\begin{array}{l} a_{20} \\ a_{21} \end{array}\right] \end{aligned}  $$

Solving (1), the weight vector, *c*, and, in turn, *a*_1_,*a*_2_ are found. From (3), it can be inferred that *w*_1_ is also expressed as a linear function of *y*_11_ or *y*_21_ alone, *i.e.*

4 $$ \begin{aligned} w_{1} = \left\{ \begin{array}{ll} &\mathrm{g}_{1} (y_{11}) = \left[\begin{array}{ll} \vec{1} & y_{11} \end{array}\right] b_{1} + \varepsilon^{\prime}_{1} = Y_{11} b_{1} + \varepsilon^{\prime}_{1} \\ &\mathrm{g}_{2} (y_{21}) = \left[\begin{array}{ll} \vec{1} & y_{21} \end{array}\right] b_{2} + \varepsilon^{\prime}_{2} = Y_{21} b_{2} + \varepsilon^{\prime}_{2} \end{array}\right. & \\ \text{where}\ b_{1} = \left[\begin{array}{l} b_{10} \\ b_{11} \end{array}\right] \!, \: b_{2} = \left[\begin{array}{l} b_{20} \\ b_{21} \end{array}\right] \end{aligned}  $$

We assume that both CCLE and GDSC sensitivity vectors maintain individual functional relationships with the latent variable, and therefore, the coefficients *a*_1_,*a*_2_,*b*_1_,*b*_2_ will remain the same for the whole response sets (*y*_1_ & *y*_2_ in Fig. 2). Using *w*_1_ and the known CCLE AUC set, *y*_21_, the coefficient *b*_2_ in (4) can be retrieved using LS minimization.

5 $$\begin{array}{*{20}l} \min_{b_{2}} \left\| w_{1} - Y_{21} b_{2} \right\|_{2}^{2} \quad \text{which results in} \; \hat{b}_{2} = Y_{21}^{+} w_{1} \end{array} $$

where (·)^+^ denotes the Moore-Penrose pseudoinverse. Using the rest of known CCLE AUC set, $\phantom {\dot {i}\!}(y_{22})_{n_{2} \times 1}$, the underlying latent vector, $\phantom {\dot {i}\!}(w_{2})_{n_{2} \times 1}$, can be retrieved following (4)

6 $$\begin{array}{*{20}l}  \hat{w}_{2} = \mathrm{g}_{2} (y_{22}) = \left[\begin{array}{ll} \vec{1} & y_{22} \end{array}\right] \hat{b}_{2} = Y_{22} \hat{b}_{2} \end{array} $$

Finally, utilizing the coefficient *a*_1_ found initially from solving (1), the unknown GDSC AUC values can be predicted following (3), as

7 $$\begin{array}{*{20}l}  \hat{y}_{12} = \mathrm{f}_{1} (\hat{w}_{2}) = \left[\begin{array}{ll} \vec{1} & \hat{w}_{2} \end{array}\right] a_{1} = \hat{W}_{2} a_{1} \end{array} $$

If only a part of CCLE drug sensitivity response is known along with a bigger portion of GDSC sensitivity set, then this whole process can be utilized for the prediction of CCLE responses by interchanging the GDSC and CCLE values.

We have also implemented a *k*NN regression based transfer learning approach for sensitivity prediction [see Additional file 1], which is computationally inexpensive to implement but often underperforms the LRP approach. We then applied an iterative update scheme to improve the performance of *k*NN approach and combined the updated *k*NN model with the LRP model [see Additional file 1]. The combined model shows similar performance to LRP model.

#### Drug sensitivity prediction via cost optimization of genomic and sensitivity data

In this section, we have utilized both gene expression and AUC data in cost optimization to improve the drug sensitivity prediction. Here, the goal is to establish a relationship between the two underlying latent variables corresponding to gene expression and AUC datasets respectively, and then exploiting this relationship for the prediction of unknown AUC values. This method is regarded as the *Latent-Latent Prediction (LLP)* since it involves the prediction of one latent variable from another. Figure 3 illustrates the use of LLP method for drug sensitivity prediction. Again, we assume that only a small portion, *y*_11_, of GDSC AUC set, *y*_1_, is known. Then, the corresponding CCLE AUC set, *y*_21_, in CCLE is used with *y*_11_ to perform the cost optimization in (1) to generate the latent vector *w*_1_ and the regression coefficients *a*_1_,*a*_2_.

Similar to the AUC optimization, the latent vector, (*v*_*k*_)_*n*×1_, corresponding to the expression vectors, $\phantom {\dot {i}\!}(x_{1k})_{n_{1} \times 1}$ & $\phantom {\dot {i}\!}(x_{2k})_{n_{1} \times 1}$ of gene *k* in GDSC & CCLE (where *k*=1,2,⋯,*p*) can be found as follows (An additional section provides the detailed development of the cost function [see Additional file 1])

8 $$\begin{array}{*{20}l}  \min_{\lambda_{k}} {\frac{\left\| x_{1k} - V_{k} \alpha_{1k} \right\|_{2}^{2} + \left\| x_{2k} - V_{k} \alpha_{2k} \right\|_{2}^{2}} {\rho(x_{1k}, v_{k}) + \rho(x_{2k}, v_{k})}}&\\ \text{subject to} \ \; \begin{array}{l} -1 \leq \lambda_{k0} \leq 1, \\ 0 \leq \lambda_{k1}, \lambda_{k2} \leq 1, \\ \lambda_{k1} + \lambda_{k2} = 1 \end{array} \end{array} $$

where $V_{k} = \left [\begin {array}{ll} \vec {1} & v_{k} \end {array}\right ]$ and $v_{k} = \left [\begin {array}{lll} \vec {1} & x_{1k} & x_{2k} \end {array}\right ] \lambda _{k}$.

Again, assuming linear relationships, $\lambda _{k} = \left [\begin {array}{lll} \lambda _{k0} & \lambda _{k1} & \lambda _{k2} \end {array}\right ]^{T}$ is the weight vector of latent *v*_*k*_ corresponding to the expression vectors *x*_1*k*_ & *x*_2*k*_, *k*-th columns of the matrices (*X*_1_)_*n*×*p*_ & (*X*_2_)_*n*×*p*_, respectively and *α*’s are the corresponding regression coefficients. The complete latent matrix, *V*_*n*×*p*_ is found performing this optimization for all *p* genes and concatenating the individual latent vectors, *i.e.*

9 $$\begin{array}{*{20}l}  V &= \left[\begin{array}{llll} v_{1} & v_{2} & \cdots & v_{p} \end{array}\right] \end{array} $$

For training, the latent matrix $\phantom {\dot {i}\!}(V_{1})_{n_{1} \times p}$ corresponding to *X*_11_ and *X*_21_ is used as model input and *w*_1_ as the corresponding output. The remaining latent, $\phantom {\dot {i}\!}(V_{2})_{n_{2} \times p}$, is utilized for prediction of the latent vector, $\phantom {\dot {i}\!}(w_{2})_{n_{2} \times 1}$. The unknown AUC values $\phantom {\dot {i}\!}(y_{12})_{n_{2} \times 1}$ are predicted using (7) again.

10 $$\begin{array}{*{20}l}  \hat{w}_{2} &= \mathcal{M}(V_{2}) \end{array} $$

11 $$\begin{array}{*{20}l} \hat{y}_{12} &= \mathrm{f}_{1} (w_{2}) = \left[\begin{array}{ll} \vec{1} & \hat{w}_{2} \end{array}\right] a_{1} = \hat{W}_{2} a_{1} \end{array} $$

We have used Random Forest (RF) [18, 20] as our prediction model here. If only a part of CCLE drug sensitivity response is known along with a bigger portion of GDSC sensitivity set, then this whole process can be utilized for the prediction of CCLE responses by interchanging the GDSC and CCLE values.

#### Combined latent drug sensitivity prediction

To improve the predictive performance of the LLP model and utilize the available CCLE data more effectively, we have incorporated the two latent variable based models together. Here, we combine the predicted latent variables from the two models *i.e.*, $w_{2}^{LRP}$ from (6) and $w_{2}^{LLP}$ from (10) via simple averaging and generate the final prediction as before.

12 $$\begin{array}{*{20}l}  \hat{w}_{2} &= \frac{\hat{w}_{2}^{LRP} + \hat{w}_{2}^{LLP}}{2} \end{array} $$

13 $$\begin{array}{*{20}l} \hat{y}_{12} &= \left[\begin{array}{ll} \vec{1} & \hat{w}_{2} \end{array}\right] a_{1} = \hat{W}_{2} a_{1} \end{array} $$

The whole process is depicted as the *Combined Latent Prediction (CLP)*. Comparisons among the three optimization based approaches yield that the combined method performs the best while the LLP approach often underperforms than LRP.

### Domain transfer approach

In this section, our goal is to analyze whether the dependency structure between CCLE and GDSC can be modeled using a common mapping across different cell lines. The hypothesis is that– if there exists such a common mapping so that the data from one domain can be shifted to the other, then the additional information available in the second database can easily be transferred to the first to produce an overall better performance [3]. For analysis, we have considered a *polynomial regression mapping* [21] and selected the polynomial order by utilizing the Spearman rank correlation (*ρ*_*s*_) between each pair of datasets from the two databases. This infers a high concordance for gene expression data between databases but poor consistency for drug sensitivity measures such as AUC or IC_50_ [5].

#### Gene expression mapping

Between GDSC and CCLE, there exist 15,664 common genes in 623 cell lines. Since the rank correlation between CCLE and GDSC gene expression is high (median *ρ*_*s*_=0.86), we have applied a gene-wise first-order polynomial regression mapping. Assume that (*g*_1*i*_)_*n*×1_ and (*g*_2*i*_)_*n*×1_ denote the expressions of the *i*-th gene in GDSC and CCLE, respectively (where *i*=1,2,⋯,*p*). Then, for each individual gene, the expression mapping from GDSC space to CCLE space

14 $$\begin{array}{*{20}l}  \hat{g}_{2i} = \alpha_{0}^{(i)} + \alpha_{1}^{(i)} g_{1i} + \varepsilon^{(i)} \end{array} $$

where $\hat {g}_{2i}$ denotes the mapped gene expression for *i*-th gene and *α*’s are the regression coefficients quantifying the strength of the association. For the total *n*×*p* gene expression matrices, the equation becomes

15 $$ {}\begin{aligned} \left[\begin{array}{llll} \hat{g}_{21} & \hat{g}_{22} & \cdots & \hat{g}_{2p} \end{array}\right] &= \left[\begin{array}{llll} \alpha_{0}^{(1)} + \alpha_{1}^{(1)} g_{11} & \alpha_{0}^{(2)} + \alpha_{1}^{(2)} g_{12} & \cdots & \alpha_{0}^{(p)} + \alpha_{1}^{(p)} g_{1p} \end{array}\right]\\ &\qquad + \left[\begin{array}{llll} \varepsilon^{(1)} & \varepsilon^{(2)} & \cdots & \varepsilon^{(p)} \end{array}\right]\\ \text{or,} \quad \hat{G}_{2} &= \stackrel{\leftrightarrow}{1} A_{0} + G_{1} A_{1} + \mathcal{E} \end{aligned}  $$

where (*A*_0_)_*p*×*p*_ and (*A*_1_)_*p*×*p*_ are two diagonal matrices containing the regression coefficients and $\mathcal {E}_{n_{1} \times p}$ is the mapping error. Here, $\stackrel {\leftrightarrow }{1}$ denotes a matrix-of-one.

16 $$\begin{array}{*{20}l}  A_{0} &= \text{diag} \! \left(\alpha_{0}^{(1)}, \alpha_{0}^{(2)}, \cdots, \alpha_{0}^{(p)} \right) \\ A_{1} &= \text{diag} \! \left(\alpha_{1}^{(1)}, \alpha_{1}^{(2)}, \cdots, \alpha_{1}^{(p)} \right) \end{array} $$

We have performed a drug-wise analysis so that only data corresponding to a single drug is available at a time. Therefore, only a subset of the common 623×15664 gene expression matrix is used for each drug, corresponding to the available cell line responses. We used ReliefF [16] to select top 200 genes from both CCLE and GDSC datasets for each drug and took the intersection as the final feature set. Figure 4 illustrates the effect of the mapping for a single gene "DBNDD1". For analysis, we have randomly selected a small subset (*i.e.*, 50 cell lines) of available GDSC samples to get the mapping from the corresponding CCLE data and evaluated the performance on the remaining cell lines. Table 5 shows the correlation between the mapped GDSC expression set with corresponding CCLE set compared to the correlation between the actual GDSC and CCLE sets for two common drugs and the mean square errors (MSE) for reconstruction. From the correlation and MSE values, it can be inferred that the mapping function successfully captures the interrelationship between CCLE and GDSC gene expression sets.

**Table 5:** Comparison of performance of gene expression mapping for two common drugs

Drug	Number of genes	Number of Test cell lines	Pearson Correlation with CCLE		Reconstruction MSE
			Original GDSC	Mapped GDSC	
17-AAG	371	259	0.8729	0.9406	0.8256
AZD6244	383	245	0.8486	0.9405	0.6297

Each result is a mean result for *n*=3 independent trials

#### Drug sensitivity mapping

For drug sensitivity measure, we used the AUC values again. The overall concordance for AUC between databases is poor (median *ρ*_*s*_=0.34), and therefore, we have considered a drug-wise second-order polynomial regression mapping. Assume that (*d*_1*j*_)_*n*×1_ and (*d*_2*j*_)_*n*×1_ denote the AUC vectors for the *j*-th drug in GDSC and CCLE, respectively. Then, for each drug, the drug sensitivity mapping from CCLE space to GDSC space

17 $$\begin{array}{*{20}l}  {}\hat{d}_{1j} = \beta_{0} + \beta_{1} d_{2j} + \beta_{2} d_{2j}^{2} + \varepsilon = D_{2j} \beta + \varepsilon, \quad \beta = \left[\begin{array}{l} \beta_{0} \\ \beta_{1} \\ \beta_{2} \end{array}\right] \end{array} $$

where $\hat {d}_{1j}$ denotes the mapped drug sensitivity dataset for *j*-th drug, $D_{2j} = \left [\begin {array}{lll} \vec {1} & d_{2j} & d_{2j}^{2} \end {array}\right ]$ is the design matrix, *β* contains the regression coefficients quantifying the strength of the association and *ε*_*n*×1_ is the mapping error.

Note that, out of the 15 common drugs, 3 of the drugs have moderate consistency (0.5≤*ρ*_*s*_<0.6) between databases, 3 have fair consistency (0.4≤*ρ*_*s*_<0.5) and the rest have poor consistency (*ρ*_*s*_<0.4). Figure 5 illustrates the effect of the mapping of AUC values from CCLE to GDSC space for the drug AZD6244 with poor consistency between databases (*ρ*_*s*_=0.26).

For analysis, again we have randomly selected 50 cell lines to get the mapping and evaluated the performance on the rest. Table 6 shows the correlation between the mapped GDSC AUC set with corresponding CCLE set compared to the correlation between the actual GDSC and CCLE sets for two common drugs and MSE for reconstruction. From the correlation and MSE values, it can be inferred that the mapping function captures the interrelationship between CCLE and GDSC drug sensitivity sets satisfactorily.

**Table 6:** Comparison of performance of drug sensitivity (AUC) mapping for two common drugs

Drug	Number of Test cell lines	Pearson Correlation with GDSC		Reconstruction MSE
		Original CCLE	Mapped CCLE	
17-AAG	259	0.5176	0.5232	0.0330
AZD6244	245	0.4022	0.3267	0.0177

Each result is a mean result for *n*=3 independent trials

#### Drug sensitivity prediction using nonlinear mapping

In this section, we have exploited the interrelationships between CCLE and GDSC through the mapping functions discussed in the previous sections. By using the mapping, we have integrated data from both databases to improve drug sensitivity prediction. Figure 6 illustrates the drug sensitivity prediction procedure using nonlinear mapping. We have performed a drug-wise analysis so that data is available for a single drug at a time. Assume that the GDSC and CCLE gene expression data are expressed as two *n*×*p* matrices, *G*_1_ and *G*_2_, respectively. Furthermore, only a small portion of *G*_1_*i.e.*, $\phantom {\dot {i}\!}(G_{11})_{n_{1} \times p}$, is available with the corresponding AUC values, $\phantom {\dot {i}\!}(d_{11})_{n_{1} \times 1}$ where *n*_1_<*n*, while the whole *G*_2_ matrix is available with the AUC response, (*d*_2_)_*n*×1_. The goal is to predict the unknown AUC values, $\phantom {\dot {i}\!}(d_{12})_{n_{2} \times 1}$, for the larger GDSC subset, $\phantom {\dot {i}\!}(G_{12})_{n_{2} \times p}$. The CCLE datasets, *G*_21_ & *d*_21_, corresponding to *G*_11_ & *d*_11_, can be utilized in this regard to acquire the individual mapping functions *h* & *f*, generated from the polynomial mapping in (15) & (17), respectively.

18 $$\begin{array}{*{20}l}  G_{21} &= h(G_{11}) = \stackrel{\leftrightarrow}{1}  A_{0} + G_{11} A_{1} \end{array} $$

19 $$\begin{array}{*{20}l}  d_{11} &= f(d_{21}) = \left[\begin{array}{lll} \vec{1} & d_{21}& d_{21}^{2} \end{array}\right] \beta = D_{21} \beta \end{array} $$

where *A*_0_,*A*_1_ are defined from (16).

To predict the AUC for *G*_12_, we map it to CCLE space using the mapping *h* as $(\hat {G}_{22})_{n_{2} \times p}$, as in Fig. 6. One can now utilize the additional information in the CCLE space by employing the complete CCLE data *G*_2_ & *d*_2_ for training the prediction model $\mathcal {M}$ while the mapped GDSC set, $\hat {G}_{22}$, is used to predict the sensitivity, $(\hat {d}_{22})_{n_{2} \times 1}$, in CCLE space. The desired prediction is then obtained by mapping it back to the GDSC space using *f*.

20 $$\begin{array}{*{20}l}  \hat{G}_{22} &= h(G_{12}) = \stackrel{\leftrightarrow}{1}  A_{0} + G_{12} A_{1} \end{array} $$

21 $$\begin{array}{*{20}l} \hat{d}_{22} &= \mathcal{M} \! \left(\hat{G}_{22} \right) \end{array} $$

22 $$\begin{array}{*{20}l}  \hat{d}_{12} &= f \! \left(\hat{d}_{22} \right) = \left[\begin{array}{lll} \vec{1} & \hat{d}_{22} & \hat{d}_{22}^{2} \end{array}\right] \beta = \hat{D}_{22} \beta \end{array} $$

The whole process is referred as the *Mapped Prediction (MP)* of GDSC data. Furthermore, if only a part of CCLE gene expression data is available with corresponding drug sensitivity values along with a bigger portion of labeled GDSC data, then this whole process can be utilized for the prediction of CCLE sensitivity by interchanging the GDSC and CCLE values. For prediction using gene expression, we have used a Bias Corrected Random Forest (BC-RF) [19, 22] model where the effect of bias correction is measured using the residual angle [23].

## Additional file

Additional file 1 Supplementary information to application of transfer learning for cancer drug sensitivity prediction. **Figure S1.** Illustration of kNN image regression prediction for unknown GDSC AUC dataset using the available CCLE data. **Figure S2.** Illustration of change in performance for a single validation set with change in the number of nearest neighbors. **Figure S3.** Illustration of prediction for a single iteration for the Updated kNN image regression prediction. **Figure S4.** Illustration of shift between GDSC and CCLE AUC distributions. **Table S1.** Comparison of MSE for reconstruction and corresponding cost function value for both optimized latent vector and mean latent vector. **Table S2.** Comparison of Pearson correlation and NRMSE among kNN Image Regression Prediction, Latent Regression Prediction and Direct Prediction of GDSC sensitivity using CCLE data. **Table S3.** Comparison of Pearson correlation and NRMSE among kNN Image Regression Prediction, Latent Regression Prediction and Direct Prediction of CCLE sensitivity using GDSC data. **Table S4.** Comparison of Pearson correlation and NRMSE among combined Latent Regression & updated kNN Image Regression Prediction, kNN Image regression Prediction, Latent Regression Prediction and Direct Prediction of GDSC drug sensitivity using CCLE data. **Table S5.** Comparison of Pearson correlation and NRMSE among combined Latent Regression & updated kNN Image Regression Prediction, kNN Image regression Prediction, Latent Regression Prediction and Direct Prediction of CCLE drug sensitivity using GDSC data. **Table S6.** Comparison of K-fold cross-validation results for 4 GDSC drug sensitivity prediction approaches using CCLE – Mapped Prediction, CCLE model Prediction, Combined Model Prediction and Direct Prediction. (PDF 648 kb)

## Abbreviations

AUC: Area under the curve
CCLE: Cancer cell line encyclopedia
CLP: Combined latent prediction
GDSC: Genomics of drug sensitivity in cancer
LLP: Latent-latent prediction
LRP: Latent regression prediction
MP: Mapped prediction
NRMSE: Normalized root mean squared error
RF: Random forest
TL: Transfer learning
