# Supplementary material for: Application of transfer learning for cancer drug sensitivity prediction
Source: BMC Bioinformatics. 2018 Dec 28;19(Suppl 17):497. doi: 10.1186/s12859-018-2465-y (PMC6309077; doi:10.1186/s12859-018-2465-y)
Supplement: Supplementary file 1 — Supplementary information to application of transfer learning for cancer drug sensitivity prediction. Figure S1. Illustration of kNN image regression prediction for unknown GDSC AUC dataset using the available CCLE data. Figure S2. Illustration of change in performance for a single validation set with change in the number of nearest neighbors. Figure S3. Illustration of prediction for a single iteration for the Updated kNN image regression prediction. Figure S4. Illustration of shift between GDSC and CCLE AUC distributions. Table S1. Comparison of MSE for reconstruction and corresponding cost function value for both optimized latent vector and mean latent vector. Table S2. Comparison of Pearson correlation and NRMSE among kNN Image Regression Prediction, Latent Regression Prediction and Direct Prediction of GDSC sensitivity using CCLE data. Table S3. Comparison of Pearson correlation and NRMSE among kNN Image Regression Prediction, Latent Regression Prediction and Direct Prediction of CCLE sensitivity using GDSC data. Table S4. Comparison of Pearson correlation and NRMSE among combined Latent Regression & updated kNN Image Regression Prediction, kNN Image regression Prediction, Latent Regression Prediction and Direct Prediction of GDSC drug sensitivity using CCLE data. Table S5. Comparison of Pearson correlation and NRMSE among combined Latent Regression & updated kNN Image Regression Prediction, kNN Image regression Prediction, Latent Regression Prediction and Direct Prediction of CCLE drug sensitivity using GDSC data. Table S6. Comparison of K-fold cross-validation results for 4 GDSC drug sensitivity prediction approaches using CCLE – Mapped Prediction, CCLE model Prediction, Combined Model Prediction and Direct Prediction. (PDF 648 kb) [file 12859_2018_2465_MOESM1_ESM.pdf]

RESEARCH

Supplementary information to application of transfer learning for cancer drug sensitivity prediction

Saugato Rahman Dhruba[1], Raziur Rahman[1], Kevin Matlock[1], Souparno Ghosh[2] and Ranadip Pal[1*]

*Correspondence: ranadip.pal@ttu.edu
[1] Department of Electrical and Computer Engineering, Texas Tech University, 1012 Boston Ave, 79409 Lubbock, TX, USA
Full list of author information is available at the end of the article

1 Latent Variable Cost Optimization Approach

1.1 Development of the Cost Function

Assume that $(y_1)_{n\times 1}$ and $(y_2)_{n\times 1}$ denote the vectors of AUC values for a particular drug in GDSC and CCLE, respectively and both can be represented as functions of a latent variable $w_{n\times 1}$ *i.e.*

$$\begin{aligned} y_1 &= \mathrm{f}_1(w) + \varepsilon_1 \\ y_2 &= \mathrm{f}_2(w) + \varepsilon_2 \end{aligned} \tag{1}$$

where ε's are $n \times 1$ error variables. From (1), it can be inferred that the vector w will also be expressed as a function of both y_1 and y_2.

$$w = \mathrm{F}(y_1, y_2) + \varepsilon \tag{2}$$

where the form of F is to be estimated by optimizing the cost function.

The appropriate cost function will contain *minimization terms* for the reconstruction errors of y_1 and y_2 from w as well as *maximization terms* for the individual concordance between pairs y_1 & w and y_2 & w. Following this, the generalized cost function and the corresponding optimization equation to estimate F can be written as

$$J = \frac{\|y_1 - \mathrm{f}_1(w)\|_2^2 + \|y_2 - \mathrm{f}_2(w)\|_2^2}{\rho(y_1, w) + \rho(y_2, w)} \tag{3}$$

$$\min_{\mathrm{F}} J = \min_{\mathrm{F}} \frac{\|y_1 - \mathrm{f}_1(w)\|_2^2 + \|y_2 - \mathrm{f}_2(w)\|_2^2}{\rho(y_1, w) + \rho(y_2, w)} \tag{4}$$

where ρ denotes a function that measures the correlation between y_i & w $(i = 1, 2)$. For our approach, we have utilized the Pearson correlation.

If we assume f_1 and f_2 as linear models for w, then from (1)

$$\begin{aligned} y_1 &= a_{10} + a_{11}w + \varepsilon_1 = \begin{bmatrix} \vec{1} & w \end{bmatrix} \begin{bmatrix} a_{10} \\ a_{11} \end{bmatrix} + \varepsilon_1 = Wa_1 + \varepsilon_1 \\ y_2 &= a_{20} + a_{21}w + \varepsilon_2 = \begin{bmatrix} \vec{1} & w \end{bmatrix} \begin{bmatrix} a_{20} \\ a_{21} \end{bmatrix} + \varepsilon_2 = Wa_2 + \varepsilon_2 \end{aligned} \tag{5}$$

where $\vec{1}_{n\times 1}$ denotes a vector-of-one *i.e.*, vector with all entries equal to unity. The coefficients a_1 and a_2 are obtained from individual Least Squares (LS) minimizations, *i.e.*

$$\min_{a_i} \|y_i - Wa_i\|_2^2 \qquad \text{which results in} \quad a_i = W^{+}y_i,\ i = 1, 2 \tag{6}$$

Following (2), w can also be expressed as a linear combination of y_1 and y_2

$$w = c_0 + c_1 y_1 + c_2 y_2 + \varepsilon = \begin{bmatrix} \vec{1} & y_1 & y_2 \end{bmatrix} \begin{bmatrix} c_0 \\ c_1 \\ c_2 \end{bmatrix} + \varepsilon = Yc + \varepsilon \tag{7}$$

where the weights satisfy the constraints $0 \leq c_1,\ c_2 \leq 1$, $-1 \leq c_0 \leq 1$ and $c_1 + c_2 = 1$.

Finally, the optimization equation in (4) can be simplified as

$$\min_c \frac{\|y_1 - Wa_1\|_2^2 + \|y_2 - Wa_2\|_2^2}{\rho(y_1, w) + \rho(y_2, w)} \quad \text{subject to} \quad \begin{array}{c} -1 \leq c_0 \leq 1, \\ 0 \leq c_1, c_2 \leq 1, \\ c_1 + c_2 = 1 \end{array} \tag{8}$$

1.1.1 Optimization of Latent Weight

We have generated and optimized the cost function in (8) for 7 common drugs between CCLE and GDSC that have responses available sufficient cell lines ($n > 200$). For cross-validation, we have utilized the $60\% - 40\%$ holdout method *i.e.*, 60% of the available cell lines are randomly used for training and the rest for testing. Table S1 illustrates the mean square errors (MSE) for reconstruction of CCLE and GDSC AUC sets from the latent vector and corresponding cost function value for 4 common drugs. We have compared these results with the case of mean latent vector *i.e.*, weight vector, $c = \begin{bmatrix} 0 & 0.5 & 0.5 \end{bmatrix}^T$. From Table S1, it is evident that the optimization scheme performs better than the mean case whenever the optimized weights are different from the mean weights. In the cases where the optimized weights ≈ 0.5 (as in AZD6244 and PD-0325901), both yield similar costs.

Table S1: Comparison of mean square error (MSE) for reconstruction and corresponding cost function value for both optimized latent vector and mean latent vector using $60\% - 40\%$ hold-out cross-validation. Each result is a mean of 3 independent trials. Bold values indicate the best performance.

Drug	Latent vector weight			Reconstruction MSE	Cost Value
	c_0	c_1	c_2		
17AAG	-0.0301	0.6411	0.3589	**0.0054**	**0.7734**
	0	0.5	0.5	0.0060	0.8153
AZD6244	-0.0995	0.5004	0.4996	**0.0059**	0.8495
	0	0.5	0.5	**0.0059**	**0.8494**
PD-0325901	-0.0995	0.5086	0.4914	**0.0061**	0.8218
	0	0.5	0.5	**0.0061**	**0.8211**
PD-0332991	-0.0935	0.7198	0.2802	**0.0022**	**0.3127**
	0	0.5	0.5	0.0028	0.3686

1.1.2 Latent Variable as Individual Functions of Datasets

From (1), if both y_1 and y_2 are represented as individual functions of w, then the latent vector can be also expressed as an individual function of either GDSC or CCLE drug response values, *i.e.*

$$w = g_1(y_1) + \varepsilon_1' = g_2(y_2) + \varepsilon_2' \tag{9}$$

where ε''s are again the error variables.

Considering the linear functional relationships in (5), g_1 and g_2 can also be inferred as linear functions, resulting in

$$\begin{aligned} w &= b_{10} + b_{11}y_1 + \varepsilon_1' = b_{20} + b_{21}y_2 + \varepsilon_2' \\ &= \begin{bmatrix} \vec{1} & y_1 \end{bmatrix} \begin{bmatrix} b_{10} \\ b_{11} \end{bmatrix} + \varepsilon_1' = \begin{bmatrix} \vec{1} & y_2 \end{bmatrix} \begin{bmatrix} b_{20} \\ b_{21} \end{bmatrix} + \varepsilon_2' \\ &= Y_1 b_1 + \varepsilon_1' = Y_2 b_2 + \varepsilon_2' \end{aligned} \tag{10}$$

where b_1 and b_2 can again be obtained from LS minimizations

$$\min_{b_j} \|w - Y_j b_j\|_2^2 \qquad \text{which results in} \quad b_j = Y_j^+ w, \quad j = 1, 2 \tag{11}$$

1.2 Analyzing Genomic Characteristics Data via Cost Optimization

1.2.1 Development of Cost Function for Genomic Data

Assume that X_1 and X_2 denote the $n \times p$ gene expression matrices for a particular drug in GDSC and CCLE, respectively. Here, both matrices can be represented as vectors containing p individual gene expression vectors, *i.e.*

$$\begin{aligned} X_1 &= \begin{bmatrix} x_{11} & x_{12} & \cdots & x_{1p} \end{bmatrix} \\ X_2 &= \begin{bmatrix} x_{21} & x_{22} & \cdots & x_{2p} \end{bmatrix} \end{aligned} \tag{12}$$

Here, $(x_{1k})_{n\times 1}$ and $(x_{2k})_{n\times 1}$ represent the expression vectors for gene $'k'$ in GDSC and CCLE, respectively. Assume that both vectors can be represented as individual functions of a latent variable $(v_k)_{n\times 1}$ corresponding to gene $'k'$. Note that, we have performed a gene-wise normalization on X_1 and X_2 to convert them to the same range of scale.

$$\begin{aligned} x_{1k} &= \varphi_1(v_k) + \varepsilon_{1k} \\ x_{2k} &= \varphi_2(v_k) + \varepsilon_{2k} \end{aligned} \tag{13}$$

where $k = 1, 2, \cdots, p$ and ε's are the error variables. Then, it can be inferred that the vector v_k will also be expressed as a function of both x_{1k} and x_{2k}.

$$v_k = \Psi_k(x_{1k}, x_{2k}) + \varepsilon_k, \quad k = 1, 2, \cdots, p \tag{14}$$

where the form of Ψ_k is to be estimated by optimizing the cost function.

Following (12), the total latent matrix for the gene expression matrices X_1 and X_2 can be written as

$$\begin{aligned} V &= \begin{bmatrix} v_1 & v_2 & \cdots & v_p \end{bmatrix} \\ &= \begin{bmatrix} \Psi_1(x_{11}, x_{21}) & \Psi_2(x_{12}, x_{22}) & \cdots & \Psi_p(x_{1p}, x_{2p}) \end{bmatrix} \end{aligned} \tag{15}$$

Similar to the AUC cost optimization in **1**, the appropriate cost function will contain minimization terms for the reconstruction errors of x_{1k} and x_{2k} from v_k and maximization terms for the correlations between x_{1k} & v_k and x_{2k} & v_k. Following this, the cost optimization equation can be written as

$$\min_{\Psi_k} \mathcal{J}_k = \min_{\Psi_k} \frac{\|x_{1k} - \varphi_1(v_k)\|_2^2 + \|x_{2k} - \varphi_2(v_k)\|_2^2}{\rho(x_{1k}, v_k) + \rho(x_{2k}, v_k)} \tag{16}$$

where ρ denotes a function that measures the correlation between x_{ik} and w $(i = 1, 2)$. Similar to AUC optimization, we have utilized the Pearson correlation here.

If we assume φ_1 and φ_2 as linear regression models for v_k for all p genes, then from (13)

$$\begin{aligned} x_{1k} &= \alpha_{10(k)} + \alpha_{11(k)} v_k + \varepsilon_{1k} = \begin{bmatrix} \vec{1} & v_k \end{bmatrix} \begin{bmatrix} \alpha_{10(k)} \\ \alpha_{11(k)} \end{bmatrix} + \varepsilon_{1k} = V_k \alpha_{1k} + \varepsilon_{1k} \\ x_{2k} &= \alpha_{20(k)} + \alpha_{21(k)} v_k + \varepsilon_{2k} = \begin{bmatrix} \vec{1} & v_k \end{bmatrix} \begin{bmatrix} \alpha_{20(k)} \\ \alpha_{21(k)} \end{bmatrix} + \varepsilon_{2k} = V_k \alpha_{2k} + \varepsilon_{2k} \end{aligned} \tag{17}$$

where α_{1k} and α_{2k} can be obtained from the individual Least square minimizations, *i.e.*

$$\min_{\alpha_{ik}} \|x_{ik} - V_k \alpha_{ik}\|_2^2 \qquad \text{which results in} \quad \alpha_{ik} = V_k^+ x_{ik}, \quad i = 1, 2 \tag{18}$$

Following (14), v_k can also be expressed as a linear combination of x_{1k} and x_{2k}

$$\begin{aligned} v_k &= \lambda_{k0} + \lambda_{k1} x_{1k} + \lambda_{k2} x_{2k} + \varepsilon_k \\ &= \begin{bmatrix} 1 & x_{1k} & x_{2k} \end{bmatrix} \begin{bmatrix} \lambda_{k0} \\ \lambda_{k1} \\ \lambda_{k2} \end{bmatrix} + \varepsilon_k = X_k \lambda_k + \varepsilon_k \end{aligned} \tag{19}$$

where the weights satisfy the same constraints again *i.e.*, $0 \le \lambda_{k1}, \lambda_{k2} \le 1$, $-1 \le \lambda_{k0} \le 1$ and $\lambda_{k1} + \lambda_{k2} = 1$. The gene-wise optimization equation in (4) can be expressed as

$$\min_{\lambda_k} \frac{\|x_{1k} - V_k \alpha_{1k}\|_2^2 + \|x_{2k} - V_k \alpha_{2k}\|_2^2}{\rho(x_{1k}, v_k) + \rho(x_{2k}, v_k)} \quad \text{subject to} \quad \begin{array}{c} -1 \le \lambda_{k0} \le 1, \\ 0 \le \lambda_{k1}, \lambda_{k2} \le 1, \\ \lambda_{k1} + \lambda_{k2} = 1 \end{array} \tag{20}$$

The complete weight matrix and regression coefficient matrices for all p genes are then

$$\begin{aligned} \Lambda &= \begin{bmatrix} \lambda_1 & \lambda_2 & \cdots & \lambda_p \end{bmatrix} \\ \mathrm{A}_1 &= \begin{bmatrix} \alpha_{11} & \alpha_{12} & \cdots & \alpha_{1p} \end{bmatrix} \\ \mathrm{A}_2 &= \begin{bmatrix} \alpha_{21} & \alpha_{22} & \cdots & \alpha_{2p} \end{bmatrix} \end{aligned} \tag{21}$$

2 Drug Sensitivity Prediction using kNN Regression

2.1 kNN Image Regression Prediction

The problem of drug sensitivity prediction can be viewed as a *missing value estimation* problem when the prediction values are already available for a number of cell lines. If the prediction values for the corresponding cell lines are already available for another database, then one can apply a transfer learning based missing value estimation approach to predict the drug sensitivities for the first database. In this section, we have utilized the kNN Impute [1] method for drug sensitivity prediction employing a secondary database, regarded as the ”kNN Image Regression Prediction”.

Figure S1 illustrates the kNN image regression approach for drug sensitivity prediction for $k = 3$ nearest neighbors. Assume that only a small portion, $(y_{11})_{n_1 \times 1}$ of GDSC drug response set, $(y_1)_{n \times 1}$ is known $(n_1 < n)$. Then, the values in the corresponding CCLE drug response set $(y_{21})_{n_1 \times p}$ are the ”images” of values in y_{11} and vice versa. Thus, the GDSC drug response set to be predicted *i.e.*, $(y_{12})_{n_2 \times 1}$ already has the CCLE image set $(y_{22})_{n_2 \times 1}$ available $(n_2 = n - n_1)$. To utilize the kNN impute method, the prediction is

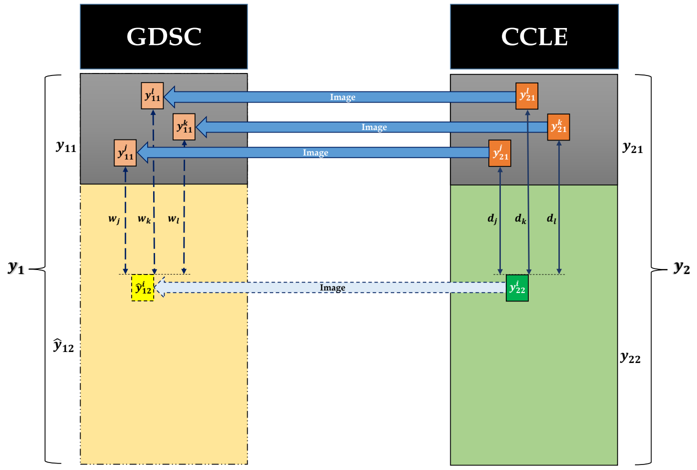

Figure S1: Illustration of kNN image regression prediction for unknown GDSC AUC dataset y_{12} using CCLE. Here, for a single value y^i_{22} from the corresponding CCLE AUC set y_{22}, $'k'$ nearest neighbors are chosen from the smaller subset y_{21}. The "images" of these neighbors in the known GDSC set y_{11} is then used to predict $\hat{y}^i_{12}$. The process is repeated for getting the whole vector, $\hat{y}_{12}$.

performed point-wise *i.e.*, only one cell line is considered at a single time. Therefore, for every point y^i_{22} in the image set y_{22}, the k nearest neighboring points in y_{21} are computed, as illustrated by 3 neighbors y^j_{21}, y^k_{21} & y^l_{21} with distances d_j, d_k & d_l in Figure S1. The "images" of these k neighbors are then located in the known GDSC set y_{11} (shown by 3 images y^j_{11}, y^k_{11} & y^l_{11} in Figure S1). The prediction value y^i_{12} is then estimated as the weighted average of the k images, where y^i_{12} is again the image of y^i_{22}.

$$\hat{y}^i_{12} = \sum_{r=1}^{k} \mathrm{w}_r y^r_{11}$$

where w_r is the weight for the r-th image neighbor y^r_{11}.

For prediction purposes, we have compared the effect of two different sets of weights – (i) weights inversely proportional to the distances ($\mathrm{w}_r \propto \frac{1}{d_r}$) and (ii) equal weights ($\mathrm{w}_r = \frac{1}{k}$).

2.1.1 Choosing Optimum k

For applying the kNN algorithm, one has to choose an optimal value of k to result in the best prediction performance. For our experiments, we have assumed that only 50 cell lines are available for GDSC (y_{11}) and the drug sensitivity prediction is performed on a larger set of cell lines ($\hat{y}_{12}$). To get the optimum k, we have performed the kNN image regression prediction on 10 different GDSC validation subsets for a particular drug for a range of k values and recorded the optimum k based on the correlation coefficient and MSE. Figure S2 illustrates the change in correlation and MSE results for different values of k for a single subset for two drugs – 17AAG and AZD6244. Here, the results from the Latent regression prediction (LRP) model are used as a baseline for performance evaluation. From Figure S2, it can be observed that both the correlation and MSE curves have a large plateau, starting from about 20 for both weight cases. Therefore, we have universally chosen $k = 25$ for our

analysis. We have also performed similar analysis for the case where CCLE drug sensitivity is predicted using GDSC values as secondary data, which results in a choice of $k = 19$. We have utilized the equal weights ("mKNN" in Figure S2) for our final analysis, since it is apparent from the figure that the equal weights case is performing better. Therefore, the predicted value is then simply the mean of the nearest neighbor images.

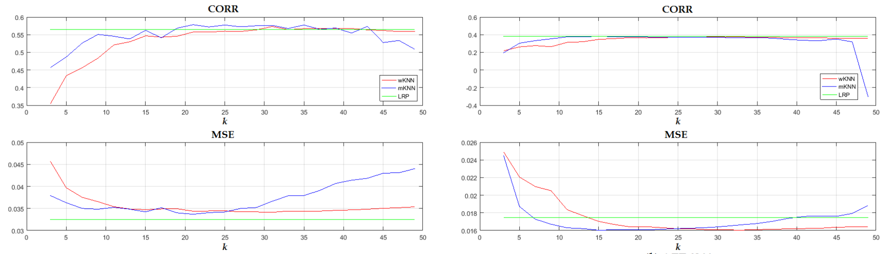

(*a*) 17AAG (*b*) AZD6244

Figure S2: Illustration of change in performance for a single validation set with change in the number of nearest neighbors, k. For prediction, the weights are either – (i) inversely proportional to the kNN distances (wKNN), or (ii) equal values for each neighbor (mKNN). Here, results from the latent regression prediction model are used as the baseline.

2.1.2 Experimental Results

We have performed the kNN Image regression for 7 common drugs between CCLE and GDSC that have responses available sufficient cell lines ($n > 200$). Table S2 shows the comparison of correlation and normalized root MSE (NRMSE) among three GDSC drug sensitivity prediction methods for 3 common drugs. Among the common cell lines between CCLE and GDSC datasets, a random subset of 50 GDSC cell lines (y_{11} in Figure S1) are used for retrieving the $k = 25$ nearest neighbor images and the remaining subset (y_{12}) is predicted along with the CCLE drug response set (y_2 in Figure S1). The first method here is the kNN image regression prediction. The second one is the Latent regression prediction. The last approach is the direct prediction of GDSC drug sensitivity set using the smaller subset for training directly. For direct prediction, we have used the intersection of top 200 ReliefF genes as the feature set. Here, each tabulated result is a mean of the results from $n = 3$ independent trials. Table S3 shows the comparison of correlation and NRMSE for the same 3 drugs when CCLE drug sensitivity values are predicted using GDSC sensitivity responses. Once again, random subsets of 50 CCLE cell lines are used for retrieving the $k = 19$ nearest neighbor images and sensitivities for the rest are predicted.

From Tables S2 & S3, it can be observed that the kNN image regression prediction method often performs better than the direct prediction in either direction (predicting CCLE sensitivities from GDSC or vice versa) as in for Nutlin-3 and PD-0332991 respectively, and for some cases, in both direction as shown for 17AAG, similar to the Latent regression prediction (LRP) method. However, this method consistently under-performs than the LRP method, which is why we considered an improved kNN image regression prediction method, as described next.

Table S2: Comparison of Pearson correlation and NRMSE among kNN Image Regression Prediction (NNP), Latent Regression Prediction (LRP) and Direct Prediction (DP) of GDSC sensitivity using CCLE data. Bold values indicate the best performance.

Drug	Pearson Correlation			NRMSE		
	NNP	LRP	DP	NNP	LRP	DP
17AAG	0.5564	**0.5602**	0.4303	0.2115	**0.2067**	0.2226
Nutlin-3	0.3085	0.4182	**0.5042**	0.1936	0.1936	**0.1804**
PD-0332991	0.2116	**0.2698**	0.0953	**0.1793**	0.1858	0.1833

Table S3: Comparison of Pearson correlation and NRMSE among NNP, LRP and DP of CCLE drug sensitivity using GDSC data. Bold values indicate the best performance.

Drug	Pearson Correlation			NRMSE		
	NNP	LRP	DP	NNP	LRP	DP
17AAG	0.4815	**0.5566**	0.2151	**0.1480**	0.1514	0.1654
Nutlin-3	0.2991	**0.4185**	0.1811	0.1415	**0.1334**	0.1432
PD-0332991	0.0423	0.2698	**0.3916**	0.2360	0.2274	**0.2164**

2.2 Drug Sensitivity Prediction via Combination of kNN Regression and Optimization

The kNN image regression prediction in **section 2** is a simple and effective model. However, the model on its own often under-performs than the more robust Latent regression prediction model or in some cases, the direct sensitivity prediction model. Therefore, in this section, we have devised a model that would improve the performance of kNN image regression model via an iterative update scheme and then combine the result with corresponding results from the LRP model to get a more robust prediction scheme.

2.2.1 Updated kNN Image Regression Prediction

For the choice of the appropriate iterative scheme, we have resorted to the renowned Gradient Descent first-order iterative optimization technique. Here, we get an initial estimate of the prediction using the kNN image regression prediction and then try to iteratively improve it through cost optimization. The cost function to be minimized here is the MSE for prediction using latent variable for the current iteration, where the prediction is defined as

$$\hat{y}_{12} = \begin{bmatrix} \vec{1} & \hat{w}_2 \end{bmatrix} a_1 = \hat{W}_2 a_1 \tag{22}$$

Here, y_{12} is the GDSC AUC set to be predicted as shown in Figure S3 and $\vec{1}$ is a vector-of-one. The value of a_1 is calculated from the cost optimization of the known sets y_{11} and y_{21}. The cost function to be optimized for updated kNN image regression is

$$\begin{aligned} \mathcal{F}\left(y_{12}^{(\tau)}, w_2^{(\tau)}\right) &= \frac{1}{n}\left\|y_{12}^{(\tau)} - \hat{y}_{12}^{(\tau)}\right\|_2^2 = \frac{1}{n}\left\|y_{12}^{(\tau)} - W_2^{(\tau)} a_1\right\|_2^2 \\ &= \frac{1}{n}\left(y_{12}^{(\tau)} - W_2^{(\tau)} a_1\right)^T \left(y_{12}^{(\tau)} - W_2^{(\tau)} a_1\right) \end{aligned} \tag{23}$$

where $y_{12}^{(\tau)}$ is the drug sensitivity response to be predicted and $W_2^{(\tau)} = \begin{bmatrix}1 & w_2^{(\tau)}\end{bmatrix}$; $w_2^{(\tau)}$ is the latent variable corresponding to $y_{12}^{(\tau)}$ & y_{22} as in Figure S3 for the τ-th iteration and defined as

$$\hat{w}_2^{(\tau)} = \begin{bmatrix}\vec{1} & y_{12}^{(\tau)} & y_{22}\end{bmatrix} c^{(\tau)} \tag{24}$$

where $c^{(\tau)}$ is the optimized latent variable weight for τ-th iteration. The gradient of the cost function in (23) w.r.t. $y_{12}^{(\tau)}$ is then

$$\nabla\mathcal{F}_{y_{12}^{(\tau)}} = \frac{2}{M}\left(y_{12}^{(\tau)} - W_2^{(\tau)} a_1\right) \tag{25}$$

The update equation following Gradient Descent algorithm would be

$$y_{12}^{(\tau+1)} = y_{12}^{(\tau)} - \gamma\frac{2}{M}\left(y_{12}^{(\tau)} - W_2^{(\tau)} a_1\right) \tag{26}$$

where γ is the growth factor, taken as a constant ($\gamma = 2$ for our analysis). The convergence criterion here is the change in prediction values between consecutive iterations.

Steps of the updated kNN image regression

I. Perform cost optimization of known sets y_{11} and y_{21}

II. Perform the kNN image regression in **section 2** to get an initial estimate of the GDSC prediction set $y_{12}^{(0)}$

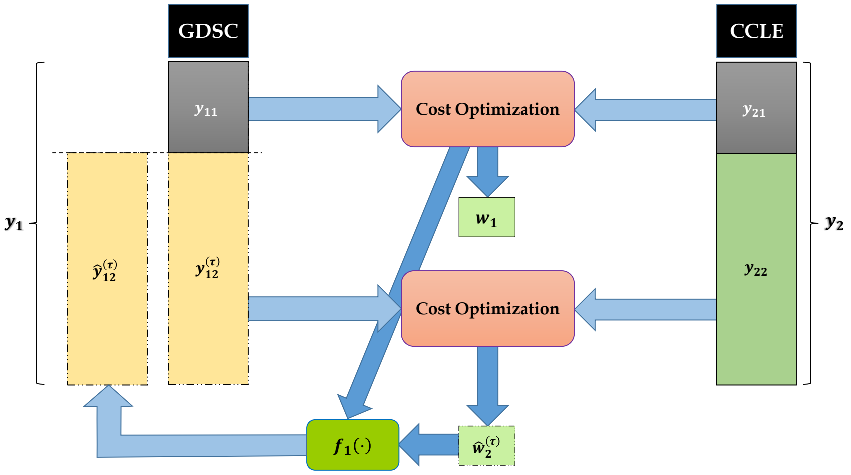

Figure S3: Illustration of prediction for τ-th iteration for the Updated kNN image regression prediction. Here, the unknown set of GDSC drug sensitivity data y_{12} is predicted using the underlying latent vector w_2 which is calculated through cost optimization with known CCLE set y_{22} and $y_{12}^{(\tau)}$, initial GDSC prediction values for n-th iteration. The final prediction for the n-th iteration is then found from (26). Here, y_{11} and y_{21} are the known set of response values that are used to perform the initial cost optimization to retrieve the functional relationships of latent with CCLE and GDSC.

III. Perform cost optimization of known set y_{22} and initial estimate $y_{12}^{(\tau)}$ for τ-th iteration to retrieve the latent vector $w_2^{(\tau)}$ using (24)

IV. Perform the prediction using (22)

V. Update the prediction using (26)

VI. if $\frac{1}{n}\left\|y_{12}^{(\tau+1)} - y_{12}^{(\tau)}\right\|_2^2 > \delta$, go to Step III

Here, δ denotes the predetermined error tolerance.

2.2.2 Combination with Latent Regression Prediction

The prediction results from Updated kNN image regression is finally combined with the prediction from Latent regression prediction (LRP) model via weighted average method. For our analysis, we have considered the weights to be equal *i.e.*

$$\hat{y}_{12} = \frac{1}{2}\left(\hat{y}_{12}^{LRP} + \hat{y}_{12}^{UNN}\right)$$

Experimental Results

We have performed the combined Latent regression and the updated kNN image regression (LRNN) prediction for 7 common drugs between CCLE and GDSC that have responses available sufficient cell lines ($n > 200$). Table S4 shows the comparison of correlation and NRMSE among 4 GDSC drug sensitivity prediction methods for 4 common drugs. As before, among the common cell lines, a random subset of 50 GDSC cell lines (y_{11} in Figure S3) are used for cost optimization in LRP and retrieval of $k = 25$ nearest neighbor images in kNN image regression. The sensitivity for the remaining subset (y_{12}) of cell lines is then predicted along with the CCLE drug response set (y_2). The first method here is the LRNN prediction described above. The remaining three are the kNN image regression prediction, Latent regression prediction and the direct prediction. For direct prediction, we have again used the intersection of top 200 ReliefF genes as the feature set. Here, each tabulated result is a mean of $n = 3$ independent trials. Table S5 shows the comparison of correlation and NRMSE for the same 4 drugs when CCLE drug sensitivity values are predicted using GDSC responses. Once again, random subsets of 50 cell lines are used for both cost optimization and retrieving the $k = 19$ nearest neighbor images and sensitivities for the rest are predicted. From Tables S4 & S5, it can be observed that for 2 of the drugs *i.e.*, 17AAG and PD-0325901, the combined LRNN prediction is performing better than the kNN image regression prediction and direct prediction methods whether the prediction is performed for missing GDSC values assuming CCLE values are known or vice versa. For the other two drugs, the LRNN prediction is performing well for only one of the cases, such as for Nutlin-3, it is only performing better when predicting for CCLE response values with GDSC responses are assumed known, while for PD-0332991, the process is performing better for predicting GDSC response with the assumption of known CCLE responses. Also, even though there is an overall significant improvement in prediction performance from the kNN image regression, the LRNN prediction still often under-performs than the Latent regression prediction. Overall, the performance of the LRNN prediction method is similar to that of the Latent regression prediction *i.e.*, out of the 7 common drugs that were tested, 5 performs better for prediction of GDSC response with the assumption of known CCLE responses, when 4 of them performs better for prediction of CCLE response with the assumption of known GDSC responses. Also, only 3 of the drugs (17AAG, Nilotinib & PD-0325901) performs better in either direction.

Table S4: Comparison of Pearson correlation and NRMSE among combined Latent Regression and the updated kNN Image Regression Prediction (LRNNP), kNN Image regression Prediction (NNP), Latent Regression Prediction (LRP) and Direct Prediction (DP) of GDSC drug sensitivity using CCLE data. Bold values indicate the best performance.

Drug	Pearson Correlation				NRMSE			
	LRNNP	NNP	LRP	DP	LRNNP	NNP	LRP	DP
17AAG	**0.5647**	0.5564	0.5602	0.4303	**0.2035**	0.2115	0.2067	0.2226
Nutlin-3	0.4114	0.3085	0.4182	**0.5042**	0.1897	0.1936	0.1936	**0.1804**
PD-0325901	0.6484	0.5465	**0.6598**	0.4225	**0.1612**	0.1775	0.1646	0.1899
PD-0332991	0.2683	0.2116	**0.2698**	0.0953	**0.1785**	0.1793	0.1858	0.1833

Table S5: Comparison of Pearson correlation and NRMSE among LRNNP, NNP, LRP and DP of CCLE drug sensitivity using GDSC data. Bold values indicate the best performance.

Drug	Pearson Correlation				NRMSE			
	LRNNP	NNP	LRP	DP	LRNNP	NNP	LRP	DP
17AAG	**0.5580**	0.4815	0.5566	0.2151	0.1509	**0.1480**	0.1514	0.1654
Nutlin-3	0.4183	0.2991	**0.4185**	0.1811	0.1422	0.1415	**0.1334**	0.1432
PD-0325901	0.6571	0.5830	**0.6586**	0.4398	0.1618	0.1821	**0.1593**	0.1928
PD-0332991	0.2629	0.0423	0.2698	**0.3916**	0.2309	0.2360	0.2274	**0.2164**

3 Combined Model Prediction

To compare the performance of the Domain Transfer (Mapped Prediction) approach presented in the main manuscript, we have implemented another transfer learning approach combining data from both CCLE and GDSC. Here, we have augmented data from the available small target dataset and the larger source dataset together to generate the training set and predicted the response for the unknown target cell lines. To explain in more details, again assume that only a portion, $(X_{11})_{n_1 \times p}$ of the whole GDSC genomic dataset, $X_{n \times p}$ $(n_1 < n)$ are available with the corresponding AUC responses, $(y_{11})_{n_1 \times 1}$, where the total dataset for CCLE, $(X_2)_{n \times p}$ is available with the corresponding AUC, $(y_2)_{n \times 1}$. For training, we have replaced the CCLE subsets $(X_{21})_{n_1 \times p}$ and $(y_{21})_{n_1 \times 1}$ with the equivalent GDSC datasets X_{11} and y_{11} (that is, the sample cell lines are the same for X_{11} & y_{11} and X_{21} & y_{21} pairs). The data for training this combined model is then

$$X_{train} = \begin{bmatrix} X_{11} \\ X_{21} \end{bmatrix}, \quad y_{train} = \begin{bmatrix} y_{11} \\ y_{21} \end{bmatrix} \tag{27}$$

while the test data is simply the gene expression set for the remaining n_2 $(= n - n_1)$ cell lines which the AUC values are assumed unknown *i.e.*, $X_{test} = X_{12}$. This model is denoted as the *Combined Model Prediction (CMP)* model.

Experimental Results

Table S6 shows the comparison of prediction performance for CMP approach for all 7 drugs with the other three methods – Mapped Prediction (MP), Direct Prediction (DP) and CCLE model prediction (CP) for K-fold cross-validation where $K = \frac{n}{50}$ and 1 fold is used for training while $(K - 1)$ folds are used for test. For prediction of AUC values

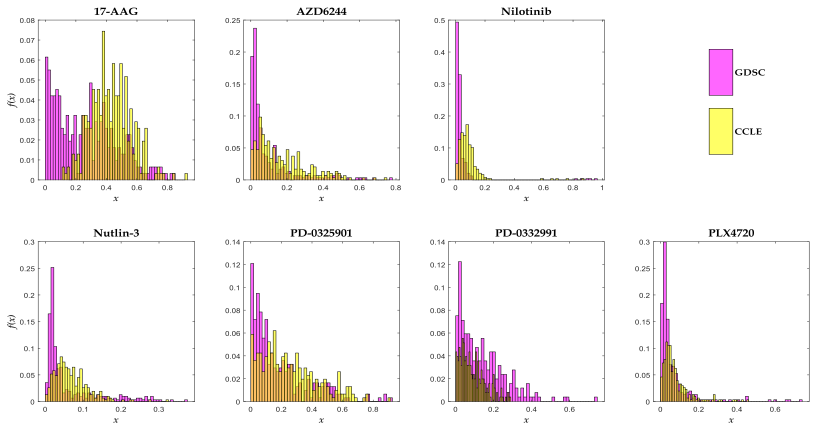

Figure S4: Illustration of shift between GDSC and CCLE AUC distributions.

using gene expression data, we have used the Random Forest model. Note that among the 7 drugs, 17-AAG and PD-0325901 have moderate concordance ($0.5 \leq \rho_s < 0.6$) while AZD6244, Nutlin-3 and PD-0332991 have poor concordance ($\rho_s < 0.4$) between databases. For PLX4720 and Nilotinib, there exist moderate to high consistency in terms of Pearson correlation ($\rho = 0.57$ and $\rho = 0.88$ respectively), although the rank correlation is low ($\rho_s = 0.29$ and $\rho_s \approx 0.1$ respectively).

Table S6: Comparison of K-fold cross-validation results for 4 GDSC drug sensitivity prediction approaches using CCLE – Mapped Prediction (MP), CCLE model Prediction (CP), Combined Model Prediction (CMP) and Direct Prediction (DP). Bold values indicate the best performance.

Drug	Pearson Correlation				NRMSE			
	MP	CP	CMP	DP	MP	CP	CMP	DP
17-AAG	**0.6062**	0.4354	0.4961	0.4591	**0.2112**	0.3073	0.2124	0.2164
AZD6244	**0.4692**	0.3580	0.4596	0.3579	0.1683	0.2173	**0.1637**	0.1743
Nilotinib	**0.8698**	0.7957	0.7876	0.4524	**0.1093**	0.1323	0.1099	0.1242
Nutlin-3	**0.5606**	0.3102	0.5133	0.5114	0.1852	0.2180	0.1854	**0.1808**
PD-0325901	**0.6132**	0.5731	0.5941	0.4224	**0.1689**	0.1875	0.1690	0.1865
PD-0332991	**0.0923**	0.0305	0.0401	0.0802	**0.1748**	0.1764	0.1761	0.1755
PLX4720	**0.6335**	0.6135	0.6104	0.5001	0.1242	0.1590	**0.1219**	0.1291

From Table S6 it can be observed that even though the MP method has the best overall correlation, in terms of NRMSE, often the CMP model performs better (*i.e.*, in 2 cases out of 7 drugs). The reason behind this lies in the distribution shift that exists between primary and secondary response distributions. Figure S4 shows the distributions of AUC values for both GDSC and CCLE for the aforementioned 7 drugs. In the cases where the primary and secondary response distributions are significantly different from each other, as in 17-AAG, the proposed Mapped prediction approach maps the primary data to secondary domain and utilizes the secondary data effectively, resulting in a significantly better prediction performance. However, in the absence of such distribution shift (as in AZD6244), the CMP approach will yield lower prediction error since the similar distributions of GDSC and CCLE response vectors effectively results in boosting the number of training samples

via combination. In the cases where the two distributions are marginally different, the MP approach will still yield a lower error, as in Nutlin-3.

Author details
[1]Department of Electrical and Computer Engineering, Texas Tech University, 1012 Boston Ave, 79409 Lubbock, TX, USA. [2]Department of Mathematics and Statistics, Texas Tech University, 1108 Memorial Circle, 79409 Lubbock, TX, USA.

References
1. Troyanskaya, O., Cantor, M., Sherlock, G., Brown, P., Hastie, T., Tibshirani, R., Botstein, D., Altman, R.B.: Missing value estimation methods for dna microarrays. Bioinformatics **17**(6), 520–525 (2001)
